# A subset of RAB proteins modulates PP2A phosphatase activity

**DOI:** 10.1038/srep32857

**Published:** 2016-09-09

**Authors:** Francesca Sacco, Anna Mattioni, Karsten Boldt, Simona Panni, Elena Santonico, Luisa Castagnoli, Marius Ueffing, Gianni Cesareni

**Affiliations:** 1 Max Plank Institute for Biochemistry, Martinried, Munich (Germany); 2 Centre for Ophthalmology, Institute for Ophthalmic Research, University of Tuebingen, Tuebingen, Germany; 3 Department DiBEST, University of Calabria, Rende, Italy; 4 Research Unit for Protein Science, Helmholtz Zentrum München, Ingolstädter Landstrasse 1, D-85764 Neuherberg, Germany

## Abstract

Protein phosphatase 2A (PP2A) is one of the most abundant serine–threonine phosphatases in mammalian cells. PP2A is a hetero-trimeric holoenzyme participating in a variety of physiological processes whose deregulation is often associated to cancer. The specificity and activity of this phosphatase is tightly modulated by a family of regulatory B subunits that dock the catalytic subunit to the substrates. Here we characterize a novel and unconventional molecular mechanism controlling the activity of the tumor suppressor PP2A. By applying a mass spectrometry-based interactomics approach, we identified novel PP2A interacting proteins. Unexpectedly we found that a significant number of RAB proteins associate with the PP2A scaffold subunit (PPP2R1A), but not with the catalytic subunit (PPP2CA). Such interactions occur *in vitro* and *in vivo* in specific subcellular compartments. Notably we demonstrated that one of these RAB proteins, RAB9, competes with the catalytic subunit PPP2CA in binding to PPP2R1A. This competitive association has an important role in controlling the PP2A catalytic activity, which is compromised in several solid tumors and leukemias.

Protein phosphatases act in concert with kinases to fine-tune signaling events by modulating the level of phosphorylated serine, threonine and tyrosine residues^1^,^2^. Protein phosphatase 2A is the most abundant serine/threonine phosphatase in mammals^3^, controlling key physiological processes, including proliferation, apoptosis, differentiation and cell migration^4^. Such broad functional specificity is mediated by the array of subunits that associate in a combinatorial fashion to form the functional PP2A holoenzyme^5^. The core enzyme is a heterodimer, formed by a catalytic subunit C (encoded by two genes, PPP2CA and PPP2CB) and a scaffold subunit A (encoded by PPP2R1A and PPP2R1B genes)^6^. The enzyme core can interact with at least 25 different regulatory subunits, resulting in more than 70 distinct trimeric complexes, differing for their subcellular localization, substrate specificity and enzyme activity^5^. Given the importance of protein-protein interactions in defining the function of PP2A, we have recently exploited an immunoprecipitation assay combined with mass spectrometry (MS)-based proteomic analysis to investigate the PP2A interactome^7^.

Besides recapitulating most of the known PP2A interactors, we found that only the scaffold subunit, and not the catalytic nor the regulatory ones, interacts with a significant number of RAB family members. RAB GTPases (Ras-related in brain) belong to the RAS superfamily of small GTPases and play a prominent role in controlling vesicle trafficking from the donor compartments to the acceptor ones^8^. Similarly to other GTPases, the RAB family members can switch from the active GTP-bound conformation, which interacts with downstream effectors proteins, to the inactive GDP-bound form^9^.

Here we report that RAB8 and RAB9 proteins interact with the PP2A scaffold subunit, PPP2R1A, in a GTP independent manner. This interaction impairs the assembly of the PP2A holoenzyme, which consequently is inactivated. Our results are consistent with a model whereby some specific members of the RAB family play a crucial role in selectively inhibiting the PP2A tumor suppressor in specific subcellular compartments.

## Results

### The PP2A holoenzyme protein interaction network

**Figure 1:**
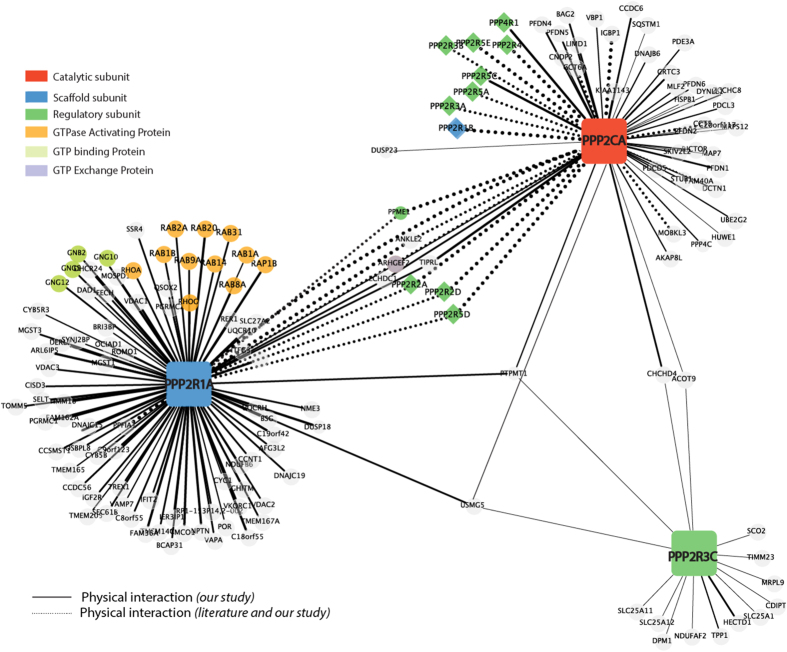
The PP2A protein interaction network. The results depicted in this figure are a subset of the results reported by Sacco *et al*.^2^. Dashed lines represent literature supported interactions, while the thickness of solid lines represent the strength of the newly identified interactions (fold change enrichment respect to the background control). Node colors depend on the functional annotation.

Protein-protein interactions play a pivotal role in defining the function of PP2A, one of the most abundant serine/threonine phosphatase implicated in cancer development. In order to investigate the PP2A interactome, we have recently exploited an immunoprecipitation assay combined with mass spectrometry (MS)-based proteomic analysis to investigate the PP2A interactome in HeLa cells. The PP2A holoenzyme protein interaction network has been investigated using transient expression and affinity purification of SF-TAG constructs of the PP2A subunits combined with MS-based proteomic analysis, as previously described^7^. The result of this approach is recapitulated as a graph in Fig. 1. As anticipated, both the scaffold and the catalytic subunit are significantly associated to many PP2A regulatory subunits (pValue < 0.003), as revealed by the DAVID functional enrichment analysis^10^. Our approach recapitulates many of the interactions already described in literature, confirming the reliability of our approach (dashed lines). As shown in Fig. 1, only PPP2R1A associates to a significant number of RAB family members (pValue < 0.0001), suggesting that such interaction may not involve the PP2A catalytic subunit. Even if a few RAB family members have been already identified as PPP2R1A interactors in Hek293 cells by large scale MS-based pull-down assays^11^, this association has not been investigated so far.

### The scaffold subunit PPP2R1A but not the catalytic subunit binds RAB8 and RAB9 *in vivo*

Among the subset of RAB family members identified as PPP2R1A interactors (Fig. 1), we decided to characterize in more detail the interaction with RAB8 and RAB9.

RAB8 mediates constitutive biosynthetic trafficking from the trans-Golgi network (TGN) to the plasma membrane, is involved in the regulation of GLUT4 vesicle translocation and in the exocytosis of a matrix metalloprotease involved in cell invasion^9^,^12^. On the other hand, RAB9 mediates tethering of late endosome-derived vesicles with the TGN and participate in the recycling of mannose-6-phosphate receptors (M6PRs)^13^.

**Figure 2:**
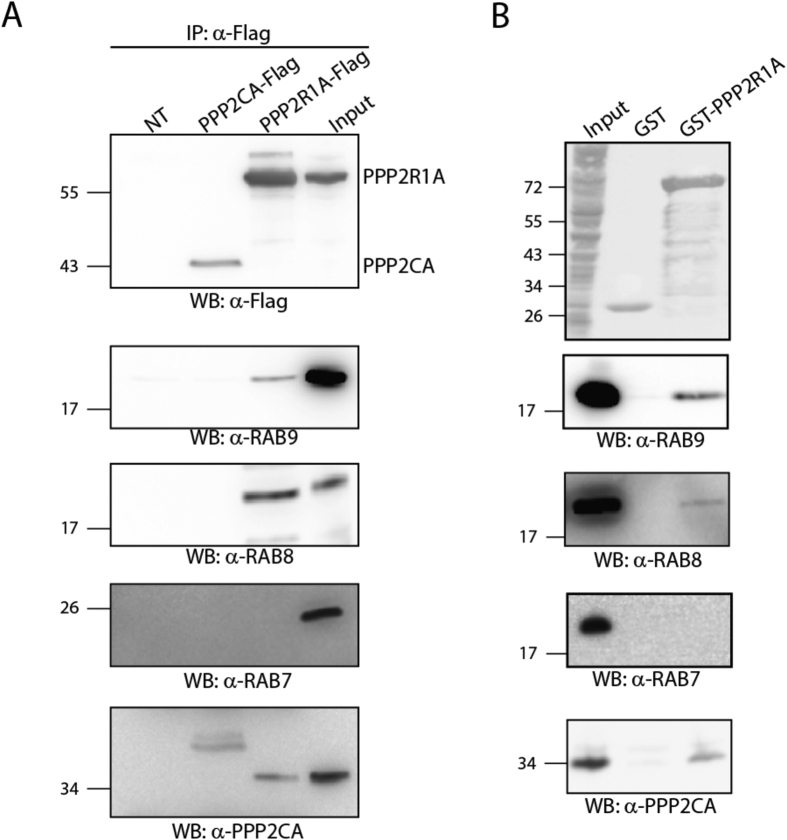
RAB proteins specifically interact *in vivo* with the PP2A scaffold subunit. (**A**) HeLa cells were transiently transfected with PPP2R1A and PPP2CA Flag constructs or with the empty vector as negative control. Protein complexes were isolated by anti-Flag beads, separated by SDS Page and incubated with anti-RAB9, anti-RAB8, anti-RAB7 and anti-PPP2CA antibodies. (**B**) HeLa cells were incubated with PPP2R1A purified as GST fusion protein and GST, as negative control. Purified complexes were separated by SDS-Page and incubated with anti-RAB9, anti-RAB8, anti-RAB7 and anti-PPP2CA antibodies.

As shown in Fig. 2A, we confirmed by co-immunoprecipitation and pull-down assays that PPP2R1A, but not PPP2CA, is able to efficiently associate with RAB8 and RAB9. Next we asked whether RAB7, a protein that was not found to be associated with PPP2R1A in our approach (Fig. 1), binds PPP2R1A either *in vivo* or *in vitro*. As shown in Fig. 1B,C, no interaction can be detected between PPP2R1A and RAB7 by both co-immunoprecipitation and pull-down assays, confirming the results of the MS-based interactomics analysis (Fig. S1).

### PPP2R1A interacts with RAB8 and RAB9 in the perinuclear region

**Figure 3:**
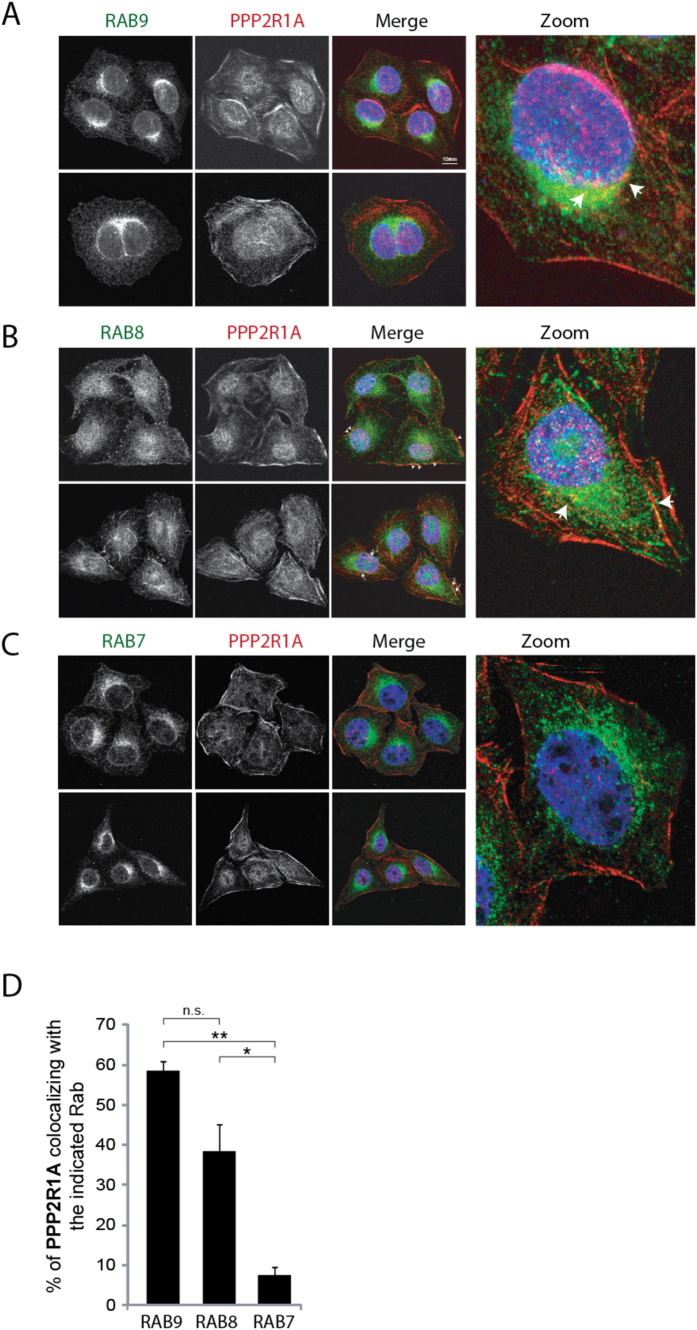
Endogenous PPP2R1A colocalizes with RAB8 or RAB9 *in vivo*. (**A–C)** Confocal microscopy of HeLa cells fixed, permeabilized and stained with anti-PPP2R1A (red) and anti-RAB7 or anti-RAB8 or anti-RAB9 (green). Yellow color indicates colocalization of the two proteins (PPP2R1A and RAB8 or PPP2R1A and RAB9). (**D**) Quantitative analysis of the percentage of colocalization was performed and results are reported in the graph as mean values ± s.e.m (see also Material and Methods). The statistical analysis was performed with a Student’s t test (n.s. = not significant).

To support the functional relevance of the observed interactions we looked at the colocalization of PPP2R1A with RAB7, as negative control, and RAB8 and RAB9 at endogenous level by performing an immunofluorescence assay. HeLa cells, chosen for their morphology, were stained with antibodies recognizing PPP2R1A and RAB7 or RAB8 or RAB9 proteins, and then analyzed by confocal microscopy. As shown in Fig. 3A, consistently with literature data, we observed that PPP2R1A displays a punctate distribution widespread throughout the cell, including the nucleus, the cytosol and the plasma membrane^3^,^14^. RAB9 occupies a discrete domain on the late endosome membranes where it controls the recycling trafficking from late endosomes to TGN^9^. In agreement with its function, we observed that RAB9 has a vesicular distribution that clusters in the perinuclear region of the cell, where it colocalizes with PPP2R1A (Fig. 3A–D). We found RAB8 localized to a system of long tubules and small vesicles scattered in the cytoplasm, and to ruffle-like structures at the plasma membrane (Fig. 3B). This distribution is consistent with its role in modulating constitutive biosynthetic trafficking from the TGN network to the plasma membrane^9^ and membrane-recycling pathway controlling protrusion formation^15^. Remarkably we demonstrated that PPP2R1A and RAB8 significantly colocalize in vesicles spread throughout the cytosol, in the plasma membrane (arrowheads) as well as in RAB8-positive tubules (arrows) (Fig. 3B–D). In agreement with our previous observation (Figs 1 and 2), we could not detect any significant colocalization between PPP2R1A and RAB7 (Fig. 3C,D).

**Figure 4:**
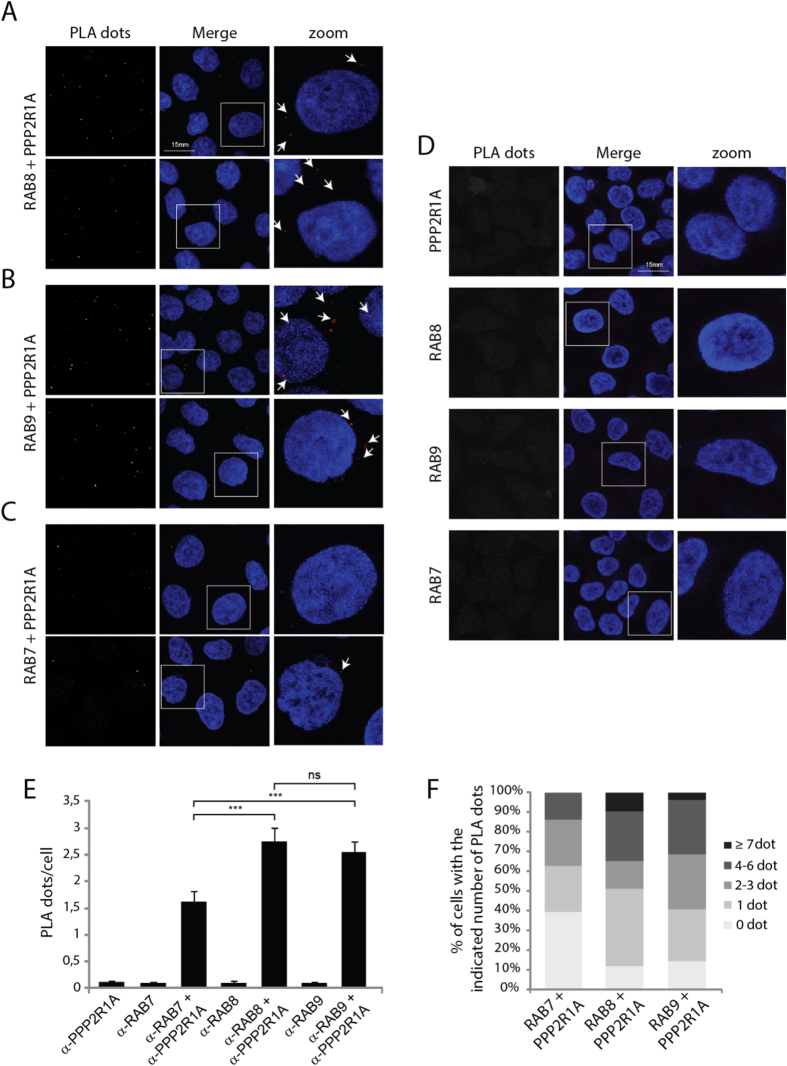
Identification of endogenous PPP2R1A and RAB8 or RAB9 complexes with *in situ* PLA. (**A–C)** HeLa cells were were fixed, permeabilized, reacted with anti-PPP2R1A and anti-RAB7 or anti-RAB8 or anti-RAB9, or with only one of these antibodies as negative controls (**D**). PLA was performed to visualize PPP2R1A-RAB interacting complexes: each red spot represents a single protein-protein interaction. Nuclei were stained with DAPI. Cells were analyzed by confocal immunofluorescence microscopy. Images for each condition are shown and representative regions were selected and magnified. The number of fluorescent red dots was quantified on at least 100 cells per condition, using the Blobfinder V3.2 software. Results are shown as mean of the number of PLA dots per cell (**E**) and as percentage of cells with the indicated number of dots (**F**). The statistical analysis was performed with a Student’s t test (n.s. =  not significant).

Next we examined the close association of PPP2R1A with RAB7, as negative control, RAB8 and RAB9 at endogenous levels using the *in situ* Proximity Ligation Assay (PLA). This technique allows monitoring interactions of endogenous proteins directly in individual cells at single-molecule resolution^16^. Significant number of proximity signals (dots) per cell was detected only in sample incubated with anti-PPP2R1A and anti-RAB8 antibodies (Fig. 4A) or with anti-PPP2R1A and anti-RAB9 antibodies (Fig. 4B–E,F), but not in the ones incubated with anti-PPP2R1A and anti-RAB7 antibodies (Fig. 4C–E,F). As negative control, the samples were incubated with each of the three antibodies (anti-PPP2R1A or anti-RAB9 or anti-RAB9 antibodies) alone and no proximity signal was detected, indicating the reliability of our experimental approach (Fig. 4D).

Taken together these observations demonstrate that PPP2R1A co-localizes with RAB8 and RAB9 in specific subcellular compartment and physically interacts with them *in situ* in cells.

### The PPP2R1A regions interacting with RAB9 and the phosphatase catalytic subunit overlap

**Figure 5:**
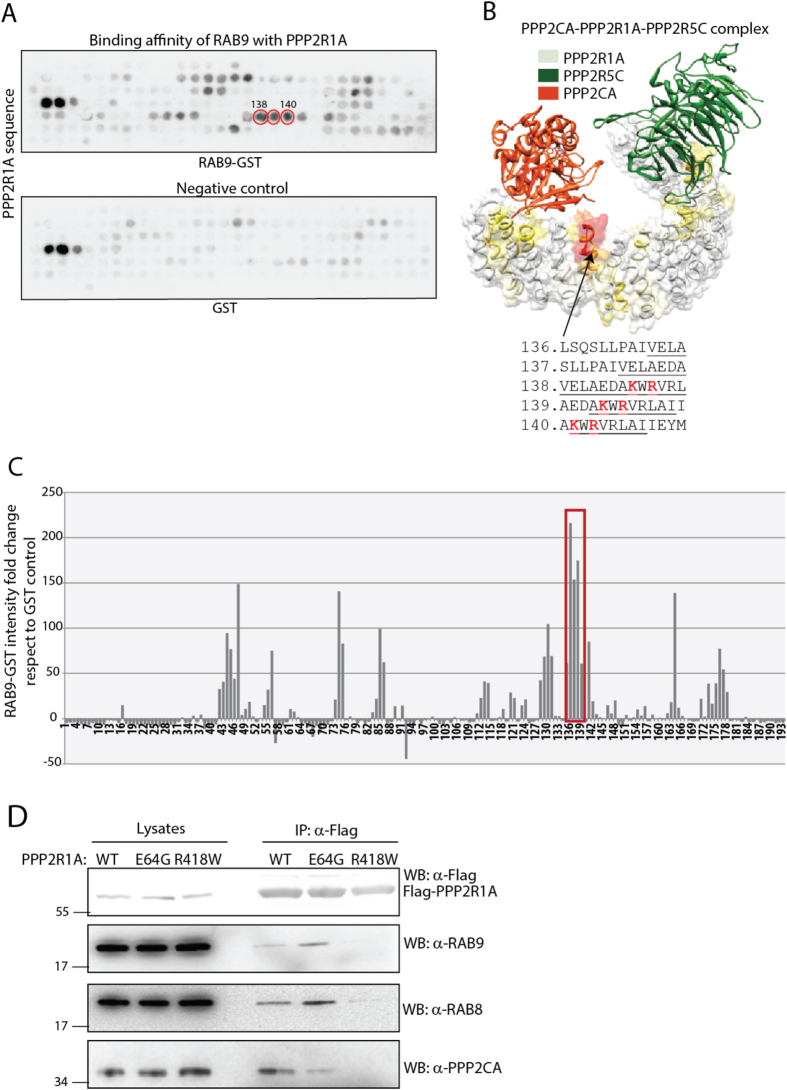
Mapping the PPP2R1A peptides that bind RAB9. (**A**) Peptide array assay. The PPP2R1A amino acids sequence, subdivided in 13mers peptides overlapping by 10 amino acids, was incubated with GST-RAB9 and GST, as negative control. The binding efficiency were revealed by incubating with an anti-GST antibody conjugated to peroxidase. The intensity of the signal was quantified and each spot was normalized on the signals of the control membrane incubated with GST (see material and methods) (**C**). (**B**) The crystal structure of the PP2A holoenzyme deposited in PDB database has been used as a scaffold to highlight the PPP2R1A regions that are bound by RAB9. PPP2R1A peptides that are bound with low affinity are white, while high affinity regions are red. Yellow and orange colors indicate intermediate affinity values. Peptides that are bound by RAB9 with the highest affinity are reported. Residues at the interface with the PP2A catalytic subunit are underlined. Residues whose mutation abolishes the PPP2CA-PPP2R1A interaction are marked in red. (**D**) HeLa cells were transiently transfected with constructs encoding the wild type form of PPP2R1A-Flag or carrying the E64G or R418W mutation. PPP2R1A protein complexes were immunoprecipitated with anti-Flag, separated by SDS-Page and revealed with anti-RAB9, anti-RAB8, anti-PPP2CA and anti-Flag antibodies.

We next aimed at mapping the contact regions on the PPP2R1A-RAB complex. 190 peptides, 13 amino acids long and spanning the entire PPP2R1A amino acid sequence, were synthesized on two nitrocellulose membranes that were incubated respectively with GST-RAB9 and GST, as negative control (Fig. 5A). The spots containing the interacting peptides were revealed by a peroxidase coupled anti-GST antibody. The results, quantified as a bar graph (Fig. 5C), show that the peptides binding RAB9 with the highest affinity map to the region of the PPP2R1A surface that interacts with the catalytic subunit [6]. Indeed RAB9 binds with the highest affinity to peptides containing Arg418 and Lys416 (Fig. 5B), whose mutation to tryptophan and glutamic acid dramatically compromise the interaction between the PP2A scaffold and the catalytic subunit^6^. To confirm these findings, we generated two PPP2R1A mutants, E64G and R418W, which respectively compromise its interaction with regulatory subunits and catalytic subunits of PP2A^17^. As expected, the R418W PPP2R1A mutant is not able to interact neither with the catalytic subunit nor with RAB8 and RAB9 proteins. On the contrary, the introduction of the E64G mutation in the PPP2R1A gene does not affect its ability to interact with PPP2CA and RAB proteins (Fig. 5D). Taken together these observations demonstrate that RAB9 and PPP2CA bind the same region of PPP2R1A, suggesting that RAB proteins and the PP2A catalytic subunit compete for interaction with the scaffold.

**Figure 6:**
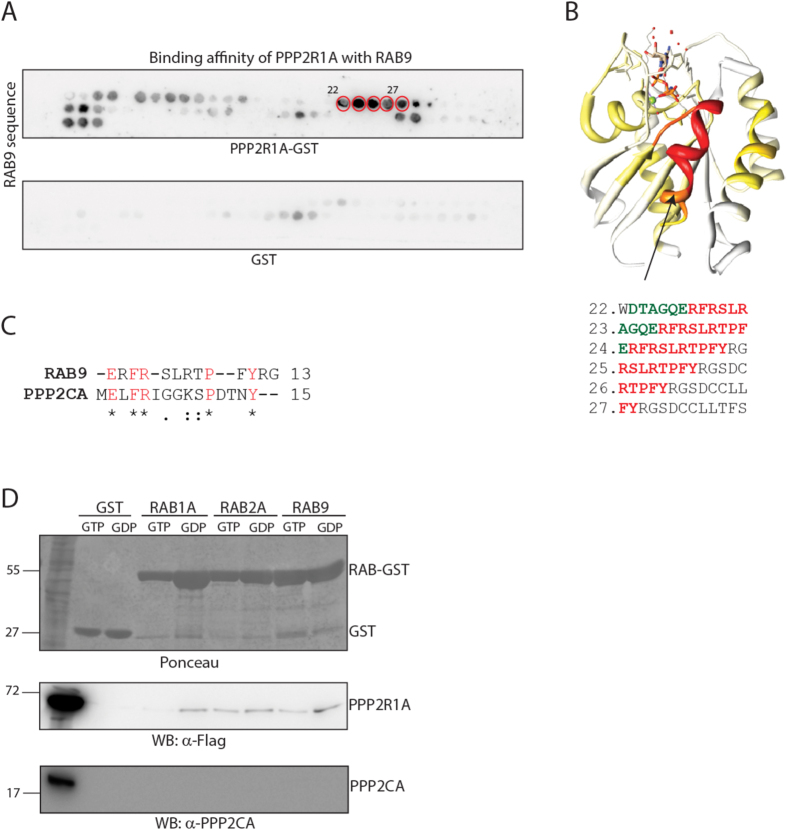
PPP2R1A binds RAB proteins in a GTP independent manner. (**A**) The RAB9 amino acids sequence was synthesized as a set of 52 overlapping 13mers. Peptides were synthesized on two cellulose membranes that were incubated with GST-PPP2R1A and GST, as negative control. (**B**) The RAB9 crystal structure deposited in PDB database has been colored according to the PPP2R1A binding affinity as determined by the SPOT synthesis analysis. High affinity regions are indicated in red, while low affinity in white. Intermediate affinity values are shown with yellow and orange colors. Peptides spot that are bound by PPP2R1A with the highest affinity are reported. Conserved nucleotide binding motif is highlighted in green, while RAB family specific residues in red. (**C**) Sequence alignment of the PPP2CA and RAB9 binding sequence with PPP2R1A. (**D**) RAB1A, RAB2A and RAB9 were purified as GST fusion proteins. RAB proteins as well as GST, as negative controls, were incubated with 0.5 mM GTPγS or 1 mM GDP. Pre-loaded RAB and GST were incubated with HeLa cells lysate over-expressing the PPP2R1A scaffold subunit. Interactors recovered by RAB proteins and GST were analyzed by SDS-Page and revealed with anti-Flag and anti-PPP2CA antibodies.

In a complementary experiment we mapped the RAB9 peptides showing affinity for PPP2R1A. The RAB9 amino acid sequence was synthesized as 13 amino acid long peptides spotted onto two nitrocellulose membranes. One membrane was incubated with GST-PPP2R1A and the other with GST, as negative control. As shown in Fig. 6A,B, PPP2R1A binds with the highest affinity six overlapping peptides, three of these containing the RAB conserved nucleotide binding motif (green). Remarkably the sequence alignment of the binding regions of RAB9 and PPP2CA revealed a partial conservation of residues mediating the contact (Fig. 6C).

### PPP2R1A binds RAB proteins in a GTP independent manner

Next we tested whether the scaffold subunit can discriminate the active (GTP bound) from the inactive (GDP bound) conformation of RAB proteins. To this aim, we analyzed the PPP2R1A association with RAB9 and other two RABs (RAB1A and RAB2A), which were previously identified by our MS-based pull-down approach, in the presence of GTPγS or GDP. As shown in Fig. S3, the PP2A scaffold subunit is efficiently purified by RAB proteins. This association is not dependent on the presence of GDP or GTP, suggesting that both the active and inactive conformations of the RAB proteins bind PPP2R1A.

### Binding of RAB9 and PPP2CA to the PPP2R1A scaffold subunit is mutually exclusive

**Figure 7:**
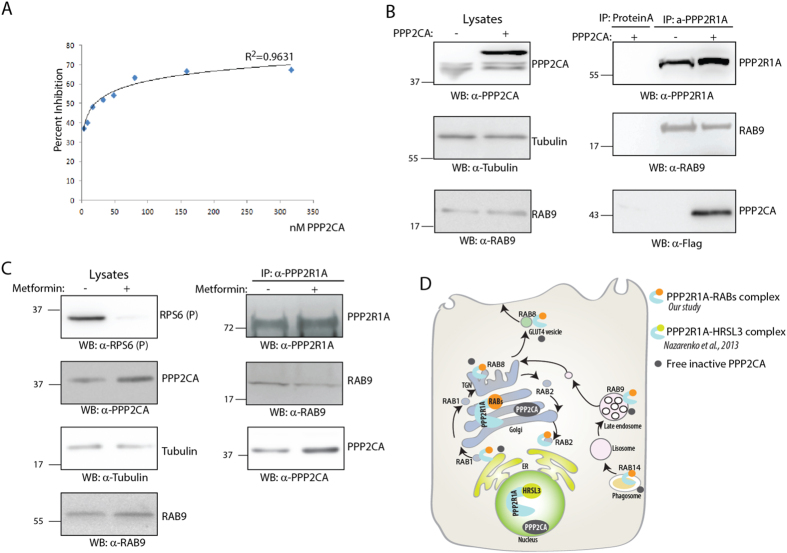
PPP2CA and RAB9 compete for the PPP2R1A binding. (**A**) Inhibition of purified PPP2R1A binding to Rab9 by PPP2CA. Recombinant PPPR1A was incubated with Rab9 0.4 μM at increasing concentration of PPP2CA (from 0.03 to 0.3 μM) The binding was measured by OD Abs (see materials and methods). (**B**) HeLa cells were transiently transfected with PPP2CA SF-TAP tagged or with the empty vector, as negative control. Lysates were analyzed by Western Blot with anti-PPP2CA, anti-RAB9 and anti-Tubulin, as loading control. PPP2R1A protein complexes were immunoprecipitated with anti-PPP2R1A, separated by SDS-Page and revealed with anti-PPP2R1A, anti-RAB9 and anti-Flag antibodies. (**C**) HeLa cells were treated with Metformin 10 mM for 24 h or left untreated. Lysates were analyzed by Western Blot with anti-phospho rpS6, anti-Tubulin, anti-RAB9 and anti-PPP2CA. Lysates were immunoprecipitated with anti-PPP2R1A antibody and the immunoprecipitated complexes were revealed with anti-PPP2R1A and RAB9 antibodies. (**D**) Schematic representation of the proposed model. The binding of RAB proteins with PPP2R1A inhibits the formation of the PP2A holoenzyme, impairing its catalytic activity.

To investigate whether RAB9 and PPP2CA compete for binding to PPP2R1A, we monitored, by competitive ELISA, the formation of the RAB9-PPP2R1A complex at increasing concentrations of the catalytic subunit PPP2CA. As shown in Fig. 7A, the higher the concentration of the catalytic subunit, the lower is the amount of RAB9 associated to PPP2R1A. This *in vitro* result is also supported by co-immunoprecipitation assays, demonstrating that the over-expression of PPP2CA significantly decreases *in vivo* the association of PPP2R1A with RAB9 (Fig. S4).

Metformin treatment increases PPP2CA expression levels^18^. We asked whether the increase in PPP2CA concentration in this condition affects the interaction of PPP2R1A with RAB9. After metformin treatment, the mTOR pathway is inactivated, as confirmed by the decrease of rpS6 phosphorylation, and the PPP2CA protein level is up-regulated (Fig. 7C, left panel). Consistent with the results in Fig. 6A,B, the increase in PPP2CA concentration is reflected by a significant reduction of the amount of RAB9 bound to PPP2R1A accompanied by a corresponding increase in the association of the catalytic and the scaffold subunits (Fig. 7C, right panel). Importantly, metformin treatment does not affect the RAB9 protein levels.

These results are consistent with a model whereby RAB9 competes with the catalytic subunit for the formation of a complex with the scaffold subunit causing the disruption of the PPP2CA-PPP2R1A dimer in distinct cell compartments.

## Discussion

In this study we report a new molecular mechanism that modulates the activity of the PP2A phosphatase in specific compartments of the cervical cancer cell line HeLa. Specifically we observe that a subset of RAB proteins compete with the PP2A catalytic subunit in binding to the scaffold subunit PPP2R1A. Such interaction disrupts the PP2A protein complex, causing its inactivation.

Co-immunoprecipitation assays as well as pull-down experiments demonstrate that PPP2R1A interacts only with a specific subset of RABs, specifically RAB8 and RAB9, but not RAB7. This observation indicates that RAB proteins likely have distinct recognition properties which determine the association with the scaffold subunit PPP2R1A. Based on our data, we are still unable to deeply describe the criteria governing the recognition of specific RAB proteins with PPP2R1A. Certainly, the recognition is not a prerogative of a specific subgroup among the six different subgroups in which the RAB protein family is subdivided^19^. In addition, we observed that RAB proteins belonging to the same subgroup, such as RAB9 and RAB7, show considerable differences in their binding efficiency towards PPP2R1A, both in co-immunoprecipitation and pull-down assays.

The association of PPP2R1A with RAB8 and RAB9 was further verified by PLAs and confocal microscopy at endogenous protein concentrations. These experiments demonstrate that both the PPP2R1A-RAB8 and PPP2R1A-RAB9 complexes occur *in vivo* in specific subcellular compartments that are determined by the endogenous distribution of the RAB protein partner. Remarkably, we were able to map the binding region of PPP2R1A and RAB9 by peptide SPOT synthesis. RAB9 binds with the highest efficiency to peptides of the PPP2R1A polypeptides that form the surface in contact with the catalytic subunit. Specifically we found that RAB9 binds the peptides containing residues Arg418 and Lys416. Mutations of these residues (R418Y and L416K) impair the activity of the phosphatase by disrupting the PPP2R1A-PPP2CA interaction and have been identified in different human cancers and^6^. Consistently with these data we show that the PPP2R1A-RAB9 interaction is decreased by the PPP2CA over-expression both *in vitro* and *in vivo*.

Since the activity and specificity of the PP2A phosphatase depends on the association of the catalytic and the scaffold subunit^10^, here we propose that RAB proteins are new regulators of the PP2A phosphatase activity (Fig. 7D). Similarly, it has been shown that HRSL3, a tumor suppressor gene, interacts with the scaffold subunit PPP2R1A, but not with the catalytic one^20^. In line with our results, the authors show that HRSL3 sequesters the catalytic subunit from the PPP2R1A protein complex, thereby inhibiting the PP2A catalytic activity. In this case, the PP2A holoenzymes involved in the interaction with HRSL3 are localized in the nucleus.

Here we propose that the disruption of the PPP2CA-PPP2R1A dimer by the competition with other regulatory proteins represent a novel molecular mechanism to regulate the catalytic activity of the PP2A holoenzyme in different subcellular compartments (Fig. 7D). The modulation of the PPP2R1A-RAB dimer abundance may play a key role in the functional inactivation of PP2A leading to cancer onset and progression^21^. Since RAB proteins have been proposed as new therapeutic targets in cancer^22^, our results suggest that the down-regulation of a subset of RAB family members in cancer tissues could restore the activity of the PP2A tumor suppressor. We expect that the results presented here will contribute to advance our understanding of the role of PP2A and RABs genes in cancer development.

## Materials and Methods

### Antibodies and Reagents

Anti-FLAG and anti-Flag M1 agarose beads were from Sigma; anti-PPP2R1A and anti-Rab8, anti-Rab9 and Rab7 antibodies were from Cell Signaling. Anti-PPP2CA from Millipore, Inc. Peroxidase-conjugated anti-rabbit, anti-mouse and anti-goat secondary antibodies were from Jackson ImmunoResearch. Antibodies for immunofluorescence: anti-Rab8 and anti Rab9 were from Cell Signaling, anti-PPP2R1A was from Santa Cruz, anti-mouse Alexa Fluor-555-conjugated secondary antibody and anti-rabbit Alexa Fluor 488-conjugated secondary antibody were from Invitrogen. PPP2CA and PPP2R3C encoding plasmids were purchased from OpenBiosystem. PPP2R1A construct was kindly provided by Marc Vidal. RAB-GST cDNAs were kindly provided by Prof. Fukuda^23^.

### Cell Culture

Cells were maintained in a humidified atmosphere at 37 °C and 5% CO2 in Dulbecco’s modified Eagle’s medium (Invitrogen), supplemented with 10% fetal bovine serum (Sigma) and 0.1% penicillin/streptomycin (Invitrogen). SILAC experiments were performed as previously described^7^. HeLa cells were transfected with Lipofectamine 2000 (Invitrogen) according to manufacturer’s protocol.

### Affinity Purification of Protein Complexes, Mass Spectrometry and Data Analysis

For one step Strep purifications, SF-TAP tagged proteins and associated protein complexes were purified essentially as described earlier^24^,^25^. HeLa cells, transiently expressing the SF-TAP tagged constructs or SF-TAP alone as control lysed in lysis buffer (containing 150 mM NaCl, 50 mM Tris-HCl, 1% Nonidet P-40, and 0.25% sodium deoxycholate, protease inhibitor cocktail (Roche) and phosphatase inhibitor cocktails II and III (Sigma-Aldrich), for 20 min at 4 °C. Lysates were incubated with Strep-Tactin-Superflow. Complexes were analyzed by MS-based proteomics as previously described^7^.

### Immunoprecipitation and Immunoblot Analysis

HeLa cells were lysed as described previously. The whole cell lysates were incubated with anti-Flag antibody conjugated to Sepharose beads over-night at 4 °C. The beads were washed with lysis buffer, and the immunoprecipitated proteins were separated by SDS-PAGE, transferred onto a nitrocellulose membrane, and immunoblotted with antibodies. The immunoreactions were visualized using ECL detection system (Amersham Biosciences).

### Pull-Down Assay

After 24 h of transfection, confluent HeLa cells were washed with ice-cold PBS and lysed in RIPA buffer (150 mm NaCl, 50 mm Tris-HCl, 1% Nonidet P-40, 0.25% sodium deoxycholate) supplemented with 1 mm pervanadate, 1 mm NaF, protease inhibitor mixture 200× (Sigma), inhibitor phosphatase mixture I and II 100× (Sigma). The samples were kept on ice for 30 min and centrifuged at 15,000 rpm at 4 °C for 30 min. The supernatant was collected, and the total amount of protein was determined by Bradford colorimetric assay (Bio-Rad). The whole cell lysates were incubated with 50 μg of the indicated GST fusion protein at 4 °C for 1 h. Thus, glutathione-Sepharose 4B beads were blocked by incubating with 3% bovine serum albumin with rocking at 4 °C for 1 h, and then after centrifugation for 3 min at 4000 × g, at 4 °C, the dry beads were bound to lysates mixed with GST fusion proteins at 4 °C for 1 h. The supernatant was discarded by centrifugation, and the beads were washed six times with lysis buffer for 3 min at 4000 × g, at 4 °C, and then the dry beads were resuspended in SDS sample buffer, boiled and analyzed by SDS-PAGE and Western blotting on nitrocellulose membrane. GST beads coupled with approximately 5 μg of the purified GST-Rabs were incubated for 20 min at 4 °C with 50 mm HEPES-KOH, pH 7.2, 150 mm NaCl, and 2.5 mm EGTA, and then MgCl2 and GTPγS (or GDP) were added to the solution at the final concentration of 10 mm and 0.5 mm (or 1 mm), respectively. The GST-Rab beads were incubated for 1 h at 4 °C with 400 μL of the cell lysates in 50 mm HEPES-KOH, pH 7.2, 150 mm NaCl, 1 mm MgCl2, 1% Triton-X-100, and protease inhibitors in the presence of 0.5 mm GTPγS (or 1 mm GDP)^23^. After washing the beads, proteins bound to the beads were analyzed by Western Blot.

### Peptides Array

Peptides of 13 amino acids were synthesized according to the standard solid phase synthesis protocols (Frank 2002), using an automatic spot synthesizer (Intavis, Koeln, Germany). The synthesis was carried out on amino-PEG membranes (Intavis). Peptides cover the whole sequences of Rab9 and PPPR1A, with a pass of 3 amino acids. The membranes were washed with PBS 1X and incubated in blocking buffer (BSA 5% PBS 1X) for 2 h at 40 C. The membranes were then incubated with 10 mg/mL dialyzed recombinant GST-Rab9 or GST-PPPR1A in blocking buffer overnight at 4 °C. After washing three times for 10 min with PBS, the anti-GST antibody was added 1/1000 in blocking buffer for 2 h at 4 °C. After three washings with PBS (10 min each), a peroxidase labeled anti-goat mAb was added 1/5000 in blocking buffer and incubated for 1.5 h at room temperature, followed by washing three times with PBS. Quantification of peptide bound domain was carried out using a chemo-luminescence substrate and the LAS-3000 instrument (Luminescent Image Analyzer, Fujifilm) instrument.

### Immunofluorescence microscopy, confocal microscopy and quantification of colocalization

HeLa cells, grown on glass coverslips, were fixed in 4% paraformaldehyde, treated with 0.1 M glycine and then permeabilized with 0.1% Triton X-100 in PBS. Cells were incubated in blocking solution (PBS-Triton 0,1%-BSA 10%), labeled with primary antibodies against the protein of interest in PBS-Triton 0,1%-BSA 10%, and lastly incubated with the specific secondary antibodies. Nuclei were stained with 4,6-diamidino-2-phenylindole (DAPI; Sigma) in PBS. Coverslips were mounted with Fluoromount Aqueous Mounting Medium (SIGMA) for observation by epifluorescence microscope. Single-plane images were acquired with a confocal laser scanner microscope (Olympus Fluoview 1000, Tokyo, Japan). Quantitative analysis of the extent of colocalization was performed by mesuring Mander’s Coefficient through the JACoP plugin^26^ of the ImageJ software (W. Rasband, National Institutes of Health, Bethesda, MDm USA). The mean ± s.e.m. percent of colocalization was calculated by analyzing a minimum of 30 cells. Statistical analysis was performed with Student’s t-test.

### *In situ* Proximity Ligation Assay (PLA)

HeLa cells were grown on glass coverslips, fixed in 4% paraformaldehyde, permeabilized with 0.1% TritonX-100-PBS1, incubated in blocking solution (BSA 10%, −0.1% Triton X-100 - PBS) for 1 h and then labeled with primary antibodies targeting PPP2R1A and Rab8 or Rab9 proteins. For the negative controls cells were labeled with only one of the two primary antibodies. Duolink *in situ* PLA was performed according to manufacturer’s protocol (Olink Bioscience, Uppsala, Sweden), as previously described^27^. PLA dots per cell were quantified with BlobFinder V3.2 image analysis software (Uppsala University, Stockholm, Sweden) and the mean number ± s.e.m of PLA dots per cell was calculated by analyzing a minimum of 100 cells. Statistical analysis was performed with Student’s t-test.

### Binding Assay

400 ng of PPP2R1A in Na2CO3 50 mM were immobilized on each microwell of an ELISA plate, 0/N at 4C. After 30 min in blocking buffer (BSA 5% in PBS1x), PPP2R1A was incubated with 100 μL of 0.4 μM Rab9 or 100 μL of 0.4 μM Rab9 plus increasing amounts of PPP2CA (from 0.03 to 0.3 μM). Wells were washed 10 times with PBS1× 0.5% Tween. Anti Rab9 (Santa Cruz) was added 1:1000 in blocking buffer for 2 h, 4 °C. After ten wash in PBS1× 0.5% Tween, a secondary antibody POD-conjugated was added for 1 h at 4 C. After washes, the binding was revealed by adding 100 μl ABTS (A3219 Sigma) and measuring optical density at 405 nm.

## Additional Information

**How to cite this article**: Sacco, F. *et al*. A subset of RAB proteins modulates PP2A phosphatase activity. *Sci. Rep.* **6**, 32857; doi: 10.1038/srep32857 (2016).

## Supplementary Material

Supplementary Information
