# Supplementary material for: A subset of RAB proteins modulates PP2A phosphatase activity
Source: Sci Rep. 2016 Sep 9;6:32857. doi: 10.1038/srep32857 (PMC5017145; doi:10.1038/srep32857)

A subset of RAB proteins modulates PP2A phosphatase activity

Francesca Sacco[1*], Anna Mattioni[1], Karsten Boldt[2], Simona Panni[3], Elena Santonico[1], Luisa Castagnoli[1], Marius Ueffing[2, 4], Gianni Cesareni[1*]

Supplementary Figures

Figure S1. Full-blots of Fig.2

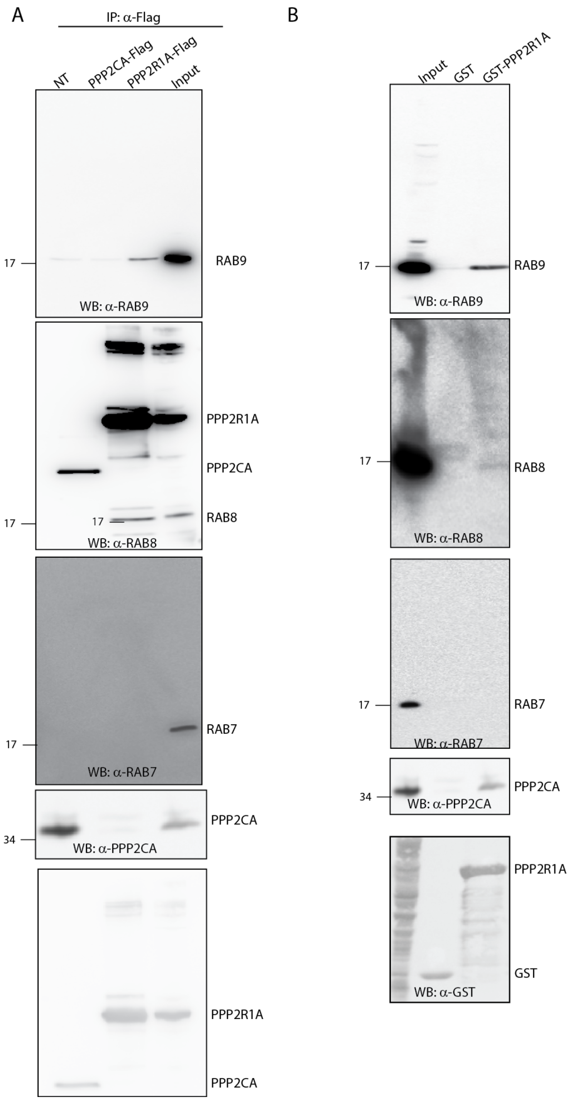

Figure S2. Full-blots of Fig.5

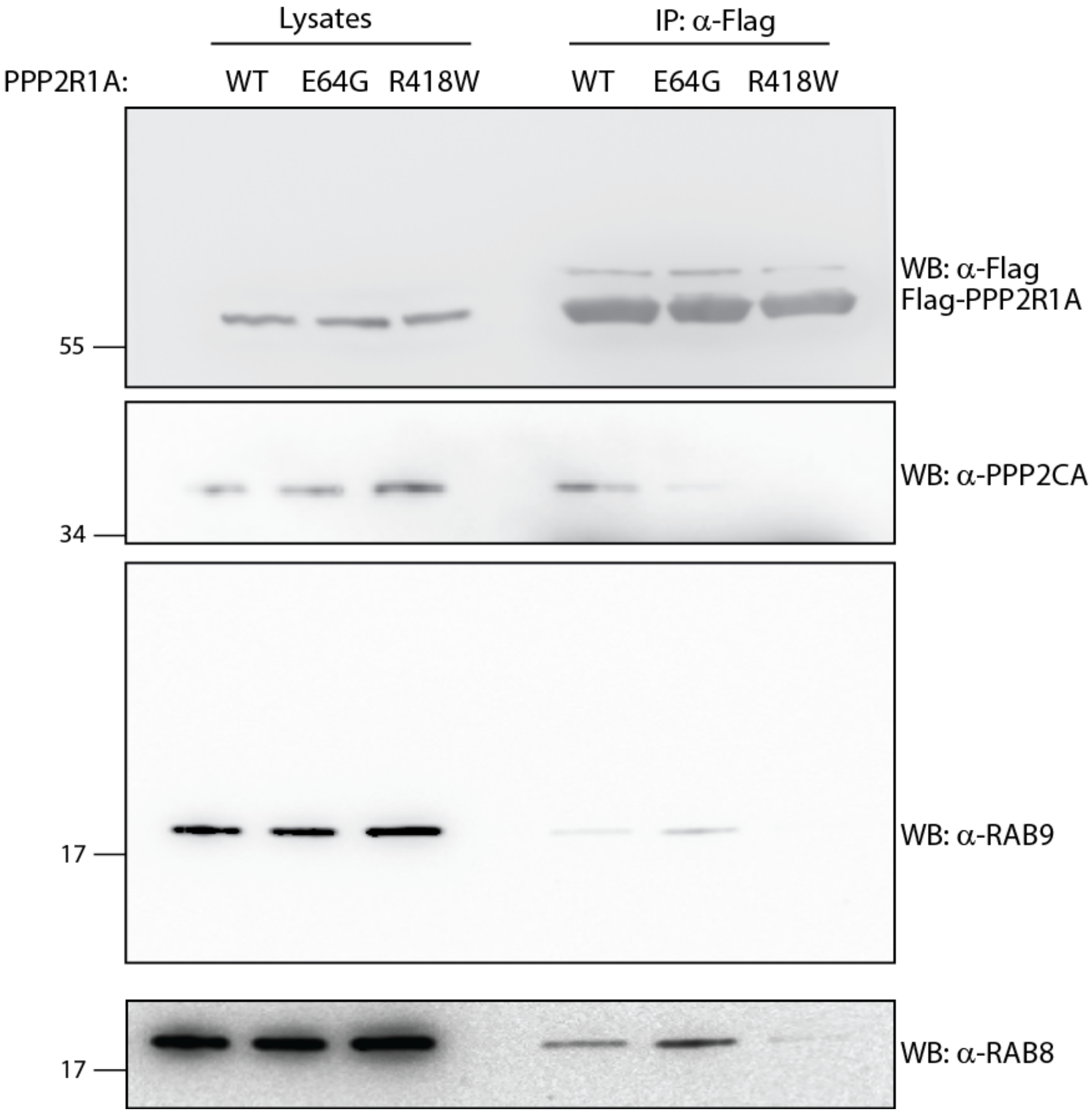

Figure S3. Full-blots of Fig.6

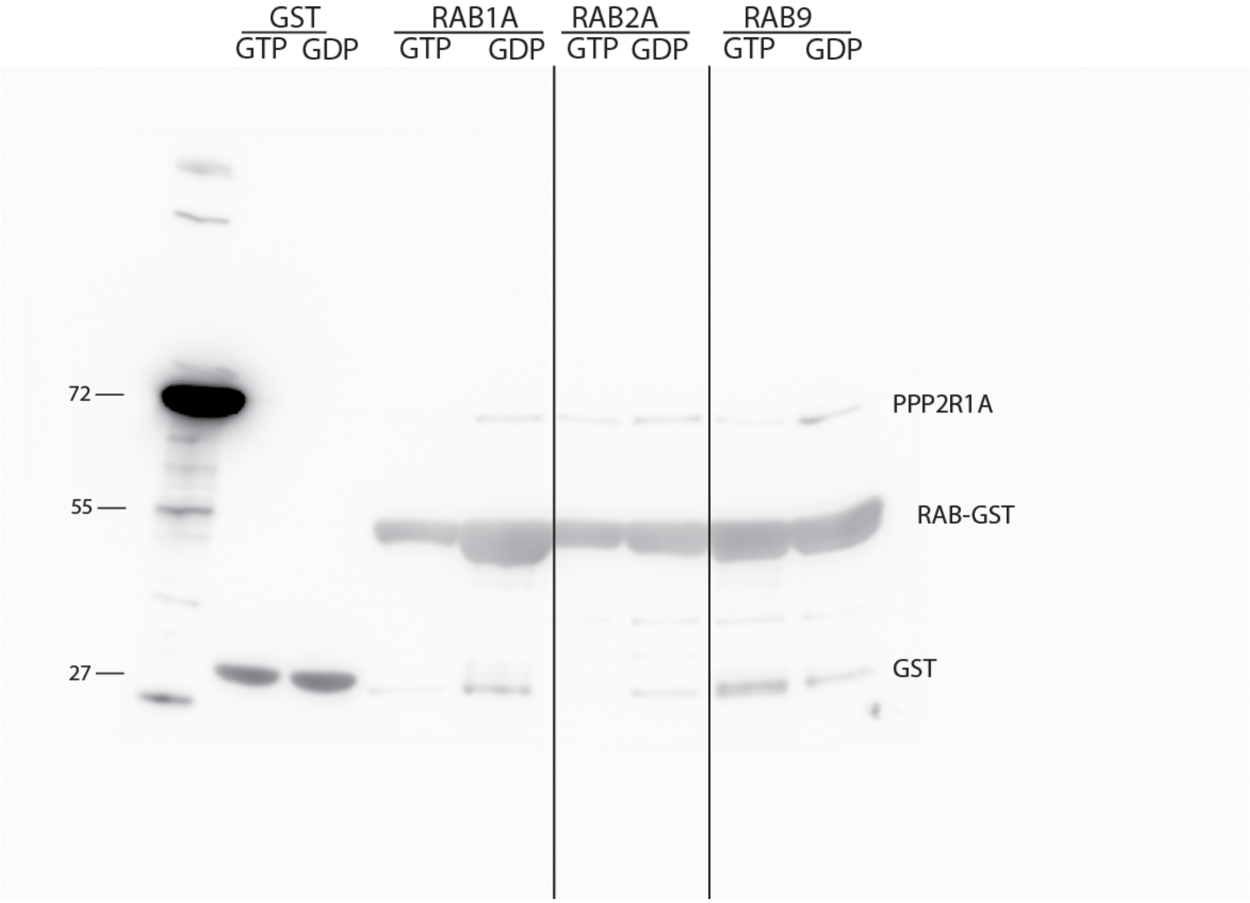

Figure S4. Full-blots of Fig.7B

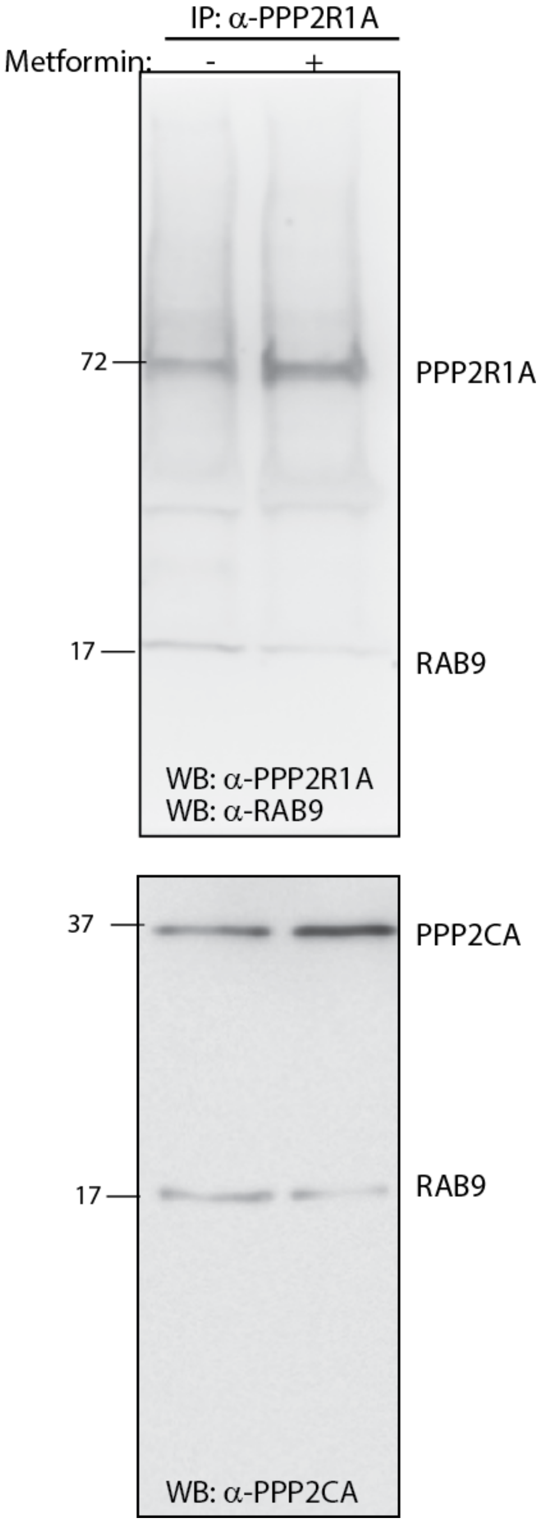

Supplement: Supplementary Information [file srep32857-s1.pdf]
